# Synergistic Effects of Multiple Factors Involved in COVID-19-dependent Muscle Loss

**DOI:** 10.14336/AD.2021.0817

**Published:** 2022-04-01

**Authors:** Nicholas Cantu, Sagar Vyavahare, Sandeep Kumar, Jie Chen, Ravindra Kolhe, Carlos M Isales, Mark Hamrick, Sadanand Fulzele

**Affiliations:** ^1^Department of Medicine, Augusta University, Augusta, GA, USA.; ^2^Department of Cell biology and anatomy, Augusta University, Augusta, GA, USA.; ^3^Division of Biostatistics & Data Sciences, DPHS, Augusta University, Augusta, GA, USA.; ^4^Department of Pathology, Augusta University, Augusta, GA, USA.; ^5^Center for Healthy Aging, Augusta University, Augusta, GA, USA

**Keywords:** COVID-19, Muscle loss, cytokine storm, glucocorticoid, antiviral drugs

## Abstract

The COVID-19 pandemic caused by the novel SARS-CoV-2 coronavirus is an ongoing pandemic causing severe health crisis worldwide. Recovered COVID-19 patients go through several long-term side effects such as fatigue, headaches, dizziness, weight loss, and muscle loss among others. Our study sought to determine the molecular mechanisms behind muscle loss in COVID-19 patients. We hypothesized that multiple factors such as cytokine storm and therapeutic drugs (glucocorticoid and antiviral drugs) might be involved in muscle loss. Using the Gene Expression Omnibus database, we identified several studies that performed RNA sequencing on skeletal muscles with the treatment of cytokine, glucocorticoid, and antiviral drugs. Our study identified cytokines, such as IL-1b, and IL-6, associated with altered regulation of several genes involved in the myogenic processes, including Ttn, Cxxc5, Malat1, and Foxo1. We also observed that glucocorticoid altered the expression of Foxo1, Lcn2, Slc39a14, and Cdkn1a. Finally, we found out that the antiviral (RNA-dependent RNA polymerase inhibitor) drug regulates the expression of some of the muscle-related genes (Txnip, Ccnd1, Hdac9, and Fbxo32). Based on our findings, we hypothesize that the cytokine storm, glucocorticoids, and antiviral drugs might be synergistically involved in COVID-19-dependent muscle loss.

The Coronavirus disease 2019 (COVID-19) is an infectious disease caused by the Severe Acute Respiratory Syndrome Coronavirus 2 (SARS-CoV-2). As an airborne virus transmissible through droplets, the spread of COVID-19 has resulted in a global pandemic that continues to put a massive strain on healthcare around the world. While the initial focus was put on the respiratory effects of COVID-19, time wide variety of side effects were noted to be associated with this novel virus across several organs. Acute infection with the virus has shown to present with a wide range of severity, and common symptoms including fever, cough, shortness of breath, and myalgias [1]. Non-respiratory effects such as headache, dizziness, abdominal pain, nausea, vomiting, and diarrhea have also been observed [1]. Pneumonia and progression to acute respiratory distress syndrome are features of severe infection along with acute cardiac injury, acute kidney injury, and death [2].

As we continue to manage patients who have recovered from acute COVID-19, observing long-term effects from infection is a major cause for concern. It is estimated that 10 to 20% of patients with symptomatic acute COVID-19 will experience persistent effects lasting greater than one month [3, 4]. Termed by some as "Long-Hauler COVID" or Post-Covid-19 Syndrome, these chronic effects include fatigue, malaise, dyspnea, headache, and inability to perform daily physical tasks [5]. Other neurological effects have also become apparent in this population, including brain fog, tremors, limb stiffness, confusion, and signs and symptoms involving cognitive functions of the brain [6].

Muscle loss, or sarcopenia, has also been demonstrated as a consequence in the COVID-19 patients [3, 7-12], but a complete explanation of the mechanisms and severity of this muscle loss has yet to be elucidated. Sarcopenia is a disease of muscle that is mainly associated with aging [11, 12]. One of the factors leading to skeletal muscle loss is the shift in equilibrium between muscle protein synthesis (MPS) and muscle protein breakdown (MPB) [12]. This balance between MPS and MPB is mediated by various factors, including decreased physical activity level, dietary protein intake, elevated level of inflammation, and oxidative stress [12].

In this study, we sought to identify possible molecular mechanisms by which muscle loss might be induced in COVID-19 patients using Gene Expression Omnibus (GEO) database and published literature. We hypothesized that a combination of several factors (such as cytokine storm (IL-1, IL-6, TNF-a), glucocorticoid, and antiviral drugs) is responsible for inducing muscle loss in COVID-19. In this study, we identified several genes differentially regulated by the factors mentioned above involved in muscle loss.

## METHODS

Through the Gene Expression Omnibus (GEO) database, we searched the published literature for studies that carried out experiments with the three elements of our hypothesis. To evaluate the effect of the cytokine storm, we searched for studies treating cells with major cytokines involved, such as IL-6 and IL-1b. We first used search terms "IL-6" and "Il-1b" along with terms like "skeletal muscle." If no studies were found on the GEO database with these criteria, we broadened the search to find studies that treated other types of cells with these molecules. A similar search strategy was employed with "dexamethasone" and "remdesivir." We included human (GEO accession; GSE10685 GSE84992,) mice model studies (GEO accession; GSE26766, GSE 74624) and cell culture studies (GEO accession; GSE23031). Details of the study design of GEO dataset are provided in Table 1. After finding studies relevant to our hypotheses, we utilized the 'GEO2R' function of GEO, allowing us to view the differential gene expression profiling between groups of our choice. After selecting the treatment and control groups, we scanned the list of differentially expressed genes to find genes relevant to the process of muscle atrophy. Furthermore, we performed Gene Ontology Enrichment Analysis for FOXO1 gene using Gene Ontology Resource software tool. All the parameters were set at default settings (Analysis Type: Panther, Annotation Version and Release Date: GO Ontology database DOI: 10.5281/zenodo.4735677 Released 2021-05-01, Analyzed List: Homo Sapiens, Reference List: Homo Sapiens-all genes in database, Annotation Set: GO biological process, Test Type: Fisher, Correction: False Discovery Rate).

**Table 1:** Details of the GEO dataset study design used for differentially expressed muscle related genes.

S. No	GEO database identification Number	Treatment/Drug	Study Design	Species/Model
1	GSE26766	IL-1β	In this study by Braun et al., IL-1β was studied with regard to its effect on the catabolism of skeletal muscle in mice (mus musculus). The mice were injected with IL-1β into the lateral ventricle of the brain. They then performed cDNA microarray analysis of mouse skeletal muscle RNA at 2, 4, and 8 hours after intracerebroventricular IL-1β injection, which yielded 494 significantly regulated genes.	Mice
2	GSE10685	IL-6	In this study by Mortensen et al., IL-6 was infused via antecubital vein for three hours into healthy young human males. Muscle biopsies were taken from the vastus lateralis and were obtained prior to infusion (0 hours), immediately after infusion (3 hours), and three hours after infusion (6 hours). Three randomly chosen subjects out of the seven total patients were selected for gene analysis. Affymetrix microarray analysis of the gene expression profile was performed for the muscle biopsies.	Human
3	GSE84992	Prednisone	In the study by Chadwick et al., Human skeletal muscle myoblasts were treated for 48 hours with various mineralocorticoid and glucocorticoid agents, one of which being prednisolone. Three replicates were treated, and another set was treated with DMSO as a vehicle (Control). RNA was isolated from the sample myoblasts, and then Affymetrix microarray was performed to determine differential gene expression.	Human Myoblast
4	GSE74624	Dexamethasone	In this study by Morrison-Nozik et al., Identify the mechanisms behind the efficacy of glucocorticoid agents in the treatment of Duchenne muscular dystrophy. In their study, they used wild-type mice as well as mice with knock out of a specific transcription factor called KLF15. The mice were treated with either dexamethasone or vehicle (4 animals in each group). Total RNA was then isolated from the quadriceps muscle of each group eight hours following treatment with either dexamethasone or vehicle. Agilent 4x44k arrays were used to show gene transcription responses. For our data, we utilized the GEO database to only analyze gene expression in the wild-type mice (dexamethasone vs. vehicle). We did not analyze data from the knock-out mouse group as we were only interested in the effect of dexamethasone itself on gene expression in skeletal muscle.	Mice
5	GSE23031	Ribavirin	In the study by Thomas et al., the anti-viral drug ribavirin was studied in order to determine its effect on gene induction in cell culture. The hepatocyte derivative cell line Huh7.5.1 was used, and three samples were treated with either ribavirin or PBS as a control. Cells were treated with ribavirin for 24 hours at a dose of 100 μg/mL. Microarray analysis and PCR assays were used to show specific genes that were induced compared to control.	Cell line: Hepatocyte derivative cell line (Huh7.5.1) Human Origin

### Results and Discussion

The overall effects of the COVID-19 pandemic have been immense, yet we still do not completely understand the issues that have and will continue to afflict patients in the long term. Sarcopenia is one such issue that can have a significant negative impact on patients, especially elderly populations. It has been well-documented that patients infected with COVID-19 are prone to developing significant weight loss and clinical cachexia [13-15]. Muscle wasting is a critical component of this weight loss and contributes to the acute and long-term effects on COVID-19 patients. A multitude of mechanisms likely contributes to this issue of muscle loss. Simply being a hospitalized patient in itself has been shown to increase the risk of significant muscle atrophy [16]. The state of immobilization inherent to being hospitalized is a major contributor to the loss of muscle mass. Loss of appetite and malnutrition are other possible impacts on these patients, as decreased amino acid intake understandably shifts the equilibrium away from muscle protein synthesis and towards muscle breakdown. Our study sought to explore some of the potential mechanisms for muscle loss that occurred at the molecular level due to changes brought on by COVID-19 infection and its typical therapeutics. Our investigation included three different mechanisms: the cytokine storm associated with COVID-19, glucocorticoid therapy, and treatment with antiviral medication.

**Figure 1.**
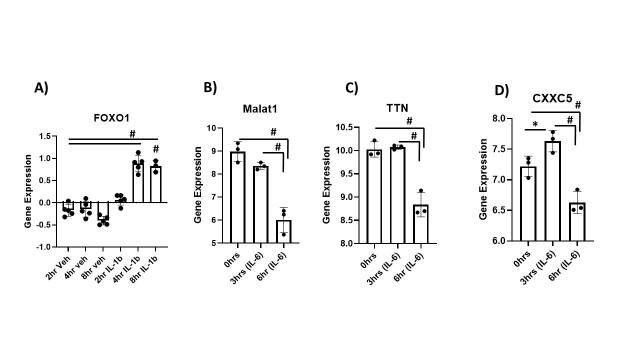
Alteration of Skeletal muscle-related genes in presence of pro-inflammatory cytokines. (A) Upregulation of Foxo1 gene expression after intracerebrovascular injection of IL-1b in skeletal muscles of mice. Foxo1 genes differentially regulated in skeletal muscle at 2 hours, 4 hours and 8 hours. The data were retrieved from GEO dataset uploaded by Braun et al. (GEO accession GSE26766) significance determined by GEO2R adjusted #P-value < 0.05. n=5. (B, C and D) Skeletal muscle-related genes differentially regulated in human skeletal muscle biopsies at 0 hours, 3 hours, and 6 hours after infusion of IL-6 compared to control. Selected genes include (B) Malat1, (C) Ttn and (D) Cxxc5. The data for (B, C and D) was retrieved from GEO dataset uploaded by Mortensen et al. (GEO accession GSE10685) significance determined by GEO2R adjusted #P-value < 0.01. n=3.

The cytokine storm associated with many viral illnesses, including COVID-19, is associated with a massive release of inflammatory cytokines (e.g., IL-6 and IL-1b). Levels of these pro-inflammatory molecules have been shown to be increased in frail and sarcopenic patients across several studies [17-19]. We searched for studies that treated skeletal muscle and other cells with some of these cytokines through the Gene Expression Omnibus database. In a study by Mortensen et al., patients were treated with IL-6 infusion, and skeletal muscle biopsies were taken before, at the start of, and after infusion [20]. MALAT1, a non-coding RNA, was downregulated with IL-6 infusion (Fig. 1). This is a long non-coding RNA (lncRNA) that has shown to be a highly-conserved and most abundantly expressed in normal tissues [21] and plays a vital role in muscle biology [22-24]. Our unpublished data (communicated) showed MALAT1 expression down-regulated with aging in skeletal muscles. Due to the link between aging and loss of muscle mass, this is the possible link between the "accelerated aging" process that seems to occur with COVID-19 infection. Titin (TTN) was also down-regulated with IL-6 treatment. Titin plays a major role in muscle contraction and generation of force required for a stable sarcomere [25]. This protein is crucial in the process of muscular hypertrophy by providing stiffness and aiding in the process of mechanosensation [26, 27]. CXXC5 is another gene that was found to be down-regulated with IL-6 treatment. This gene encodes for a zinc-finger protein that has a role in signal transduction as a transcription factor and epigenetic regulator [28]. Notably, CXXC5 has been shown to regulate the differentiation of C2C12 myoblasts into myocytes and thus plays a critical role in the growth of skeletal muscle [29].

IL-1b is another major player involved in the cytokine storm. In a study by Braun et al., IL-1b was injected into the mouse cerebral ventricles to determine its role in signaling in the central nervous system and the effect on skeletal muscle catabolism [30]. Significant upregulation of Foxo1 was seen at 4 and 8 hours of IL-1b infusion (Fig. 1). This gene encodes for a transcription factor in the forkhead box family, which acts directly on the muscle-specific E3 ubiquitin ligases. It has been shown to be up-regulated in skeletal muscle during atrophy, delay muscle regeneration and suppress myoblast proliferation, making it one of the primary players responsible for muscle atrophy [31-33].

**Figure 2.**
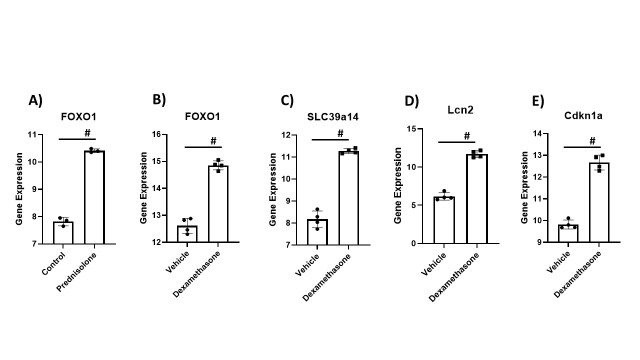
Alteration of Skeletal muscle-related genes in presence of glucocorticoid (A) Foxo1 gene expression in human skeletal muscle myotubes treated with prednisolone compared to control. The data for (A) was retrieved from GEO dataset uploaded by Chadwick et al. (GEO accession GSE84992) significance determined by GEO2R adjusted #P-value < 0.01. n=3. (B-E) Skeletal muscle-related genes differentially regulated with treatment of mice with dexamethasone compared to control, including B) Foxo1, C) Slc39a14, D) Lcn2 and (E) Cdkn1a. The data was retrieved from GEO dataset uploaded by Morrison-Nozik et al. (GEO accession GSE 74624) significance determined by GEO2R adjusted #P-value < 0.01 n=4.

Next, we analyzed studies involving glucocorticoid agents, as the anti-inflammatory effect of these medications was thought to prevent progression to respiratory failure and death in COVID-19 [34]. In a study from 2017 by Chadwick et al., gene expression changes of human primary skeletal muscle myotubes treated with prednisolone vs. control were analyzed [35]. The Foxo1 gene was up-regulated by prednisolone treatment (Fig. 2). A study on dexamethasone-treated skeletal muscle in mice also showed up-regulation of the Foxo1 gene compared to vehicle-treated mice [36], further supporting that glucocorticoids may play a role in muscle atrophy. We hypothesize that Foxo1 gene might be synergistically (cytokines and glucocorticoid treatment) up-regulated in Covid19 patients experiencing muscle loss. In-depth analysis of the Foxo1 gene using Gene Ontology Enrichment analysis revealed that Foxo1 is involved in six biological processes associated with aging, nine biological processes associated with senescence, and three hundred eighty-four biological processes associated with muscle (Supplementary Table 1). We also identified several other genes of interest that were up-regulated, including Lcn2, Slc39a14, and Cdkn1a, with dexamethasone treatment (Fig. 2). A recent study in mice argues that LCN2 functions as a potent pro-inflammatory factor in skeletal muscle response to obesity-related sarcopenia and is thus a therapeutic candidate target for sarcopenia treatment [37]. Slc39a14 belongs to a family of metal transporters and plays a role in zinc uptake in muscle progenitor cells [38]. It has been previously reported that up-regulation of this gene plays a role in metastatic cancer-related cachexia by altering zinc homeostasis, which can repress myogenic factor expression and induce myosin heavy chain loss [39]. Cdkn1a (also known as p21) has long been known for its role in many cellular processes (e.g., cell differentiation, autophagy, and senescence), and its expression was elevated with dexamethasone treatment. Muscle atrophy induced by limb immobilization is mediated by p21, as this is a downstream effector of the gene p53, which controls cell division and cell death [40]. A study by Fox et al. provides evidence that Cdkn1a induces muscle atrophy without immobilization [40]. This opens the door for yet another mechanism by which glucocorticoid administration might be involved in muscle loss.

**Figure 3.**
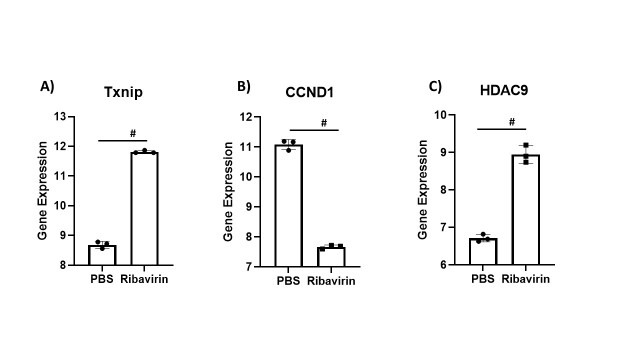
Alteration of Skeletal muscle-related genes in the presence of antiviral drug Rabavirin (A) Skeletal muscle-related genes differentially regulated with ribavirin treatment of Huh7.5.1 cells. (A) Txnip, (B) Ccnd1 and (C) Hdac9. The data was retrieved from GEO dataset uploaded by Thomas et al. (GEO accession GSE23031) significance determined by GEO2R adjusted #P-value < 0.01. n=3.

Lastly, we searched for evidence that antiviral agents may play a role in muscle loss, as the drug remdesivir has been used across the world in the treatment of patients with COVID-19. Due to the lack of studies found on the Gene Expression Omnibus analyzing remdesivir's effect on cellular gene expression, we looked for other antiviral drugs with a similar mechanism (RNA-dependent RNA polymerase inhibitor). Ribavirin is another RNA-dependent RNA polymerase inhibitor and is currently used as a therapeutic for the hepatitis C virus. This drug works as a nucleoside analog and binds the RNA polymerase enzyme at the same binding site as remdesivir [41]. Thomas et al. (2012) treated primary hepatocytes (Huh7.5.1) with ribavirin and phosphate-buffered saline (PBS) to explore ribavirin's mechanisms of action and induction of various genes [42]. The gene Thioredoxin-interacting protein (Txnip) was up-regulated with ribavirin compared to PBS (Fig. 3). Txnip gene plays a vital role in energy metabolism, including reducing skeletal muscle insulin sensitivity [43]. Hdac9, a histone deacetylase (HDAC) gene, was also shown to be up-regulated with ribavirin in this study (Fig. 3). Acetylation level of histones is a major regulator of DNA transcription, and more recently, HDACs have been demonstrated to control muscle differentiation [44]. An increase in Hdac9 specifically has exhibited an ability to suppress myoblast differentiation [45].

Cyclin D1 (CCND1) is an important member of the cyclin family of genes which was downregulated with ribavirin treatment (Fig. 3). Skeletal muscle stem cells, which aid in the process of muscle repair, have been shown to lose the ability to express CCND1 with age [46]. Thus, treatment with antivirals like ribavirin may also negatively affect patients' ability to repair muscle. We did find data from a study treating Vero E6 cells with remdesivir to determine changes in gene expression [47]. CCND1 was downregulated in the remdesivir treated cells, as was demonstrated by ribavirin in the previous study. Fbxo32, the gene for a protein called MAFBx, displayed increased expression in the Vero cells treated with remdesivir. MAFBx and its counterpart MuRF1 (Trim63) make up the E3 ubiquitin ligase system [48] that is up-regulated in muscle atrophy conditions, including dexamethasone treatment [49] and interleukin-1-induced cachexia [50]. Upregulation of this gene by remdesivir poses the possibility that treatment with this drug may also contribute to muscle atrophy.

**Figure 4.**
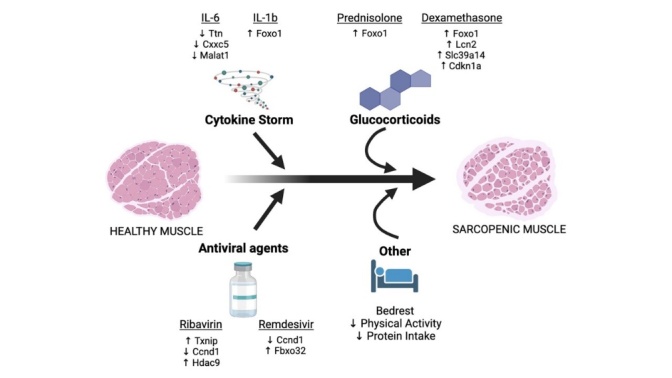
Possible molecular mechanism involved in COVID-19-dependent muscle loss (created using BioRender.com). The cytokines like IL-1b and IL-6 were associated with the altered regulation of several genes involved in the myogenic processes (Ttn, Cxxc5, Malat1, and Foxo1). We also found glucocorticoid alters the expression of Foxo1, Lcn2, Slc39a14, and Cdkn1a. The antiviral (RNA-dependent RNA polymerase inhibitor) drug regulates the expression of some of the muscle-related genes (Txnip, Ccnd1, Hdac9, and Fbxo32). Synergistic effect of these multiple factors might be involved in COVID-19-dependent muscle loss.

Our study does have some inherent limitations. We identified only a selected number of genes affected by the various treatments that play a role in muscle biology. These are likely only a fraction of the total number of genes implicated in sarcopenia as a whole. Secondly, we could not study gene expression changes in human skeletal muscle cells across all of these studies. Another important factor to consider is our inability to determine gene expression that COVID-19 infection itself has on skeletal muscle in combination with glucocorticoids and antiviral medications. In the future, further gene sequencing of human skeletal muscle cells infected by the coronavirus in combination with dexamethasone and remdesivir treatment is needed. This would likely provide a more accurate idea of the genetic mechanisms behind sarcopenia in COVID-19 patients. In summary, we found several differentially regulated gene expression that occurs when cells are treated with cytokines, glucocorticoids, and antivirals. These changes present the possibility that muscle atrophy may be induced by these conditions separately or synergistically. In patients infected with severe COVID-19 with long-term symptoms, it is likely they have experienced the cytokine storm as well as treatment with glucocorticoids like dexamethasone and the antiviral drug. Based on our findings, we speculate that an additive effect of these three factors might involved in muscle loss (Figure.4). Due to the deleterious effects of muscle loss on morbidity and mortality, careful consideration for these effects is needed when treating COVID-19 patients in the future.

## Supplementary Materials

The Supplementary data can be found online at: www.aginganddisease.org/EN/10.14336/AD.2021.0817.


