# Supplementary material for: Synergistic Effects of Multiple Factors Involved in COVID-19-dependent Muscle Loss
Source: Aging Dis. 2022 Apr 1;13(2):344–52. doi: 10.14336/AD.2021.0817 (PMC8947833; doi:10.14336/AD.2021.0817)
Supplement: Supplementary file 1 [file AD-13-2-344-s.pdf]

SUPPLEMENTARY DATA

Synergistic Effects of Multiple Factors Involved in COVID-19-dependent Muscle Loss

Cantu Nicholas[1], Sagar Vyavahare[2], Sandeep Kumar[2], Jie Chen[3], Ravindra Kolhe[4], Carlos M. Isales[1,5] Mark Hamrick[2,5], Sadanand Fulzele[1,2,5]*

© 2021. Cantu N et al. Published online at http://www.aginganddisease.org/EN/10.14336/AD.2021.0817

SUPPLEMENTARY DATA

Supplementary Table 1. Gene Ontology Enrichment analysis of Foxo1 gene.

GO biological process complete	Homo sapiens - REFLIST (20595)	upload_1 (1)	upload_1 (expected)	upload_1 (over/under)	upload_1 (fold Enrichment)	upload_1 (raw P-value)	upload_1 (FDR)
**AGING**							
aging (GO:0007568)	296	0	0	-	< 0.01	1.00E+00	1.00E+00
positive regulation of cell aging (GO:0090343)	16	0	0	-	< 0.01	1.00E+00	1.00E+00
negative regulation of cell aging (GO:0090344)	26	0	0	-	< 0.01	1.00E+00	1.00E+00
multicellular organism aging (GO:0010259)	35	0	0	-	< 0.01	1.00E+00	1.00E+00
regulation of cell aging (GO:0090342)	51	0	0	-	< 0.01	1.00E+00	1.00E+00
cell aging (GO:0007569)	76	0	0.01	-	< 0.01	1.00E+00	1.00E+00
**SENESCENCE**							
animal organ senescence (GO:0010260)	3	0	0	-	< 0.01	1.00E+00	1.00E+00
oncogene-induced cell senescence (GO:0090402)	3	0	0	-	< 0.01	1.00E+00	1.00E+00
oxidative stress-induced premature senescence (GO:0090403)	4	0	0	-	< 0.01	1.00E+00	1.00E+00
stress-induced premature senescence (GO:0090400)	8	0	0	-	< 0.01	1.00E+00	1.00E+00
positive regulation of cellular senescence (GO:2000774)	14	0	0	-	< 0.01	1.00E+00	1.00E+00
replicative senescence (GO:0090399)	16	0	0	-	< 0.01	1.00E+00	1.00E+00
negative regulation of cellular senescence (GO:2000773)	20	0	0	-	< 0.01	1.00E+00	1.00E+00
regulation of cellular senescence (GO:2000772)	44	0	0	-	< 0.01	1.00E+00	1.00E+00
regulation of cellular senescence (GO:2000772)	44	0	0	-	< 0.01	1.00E+00	1.00E+00
cellular senescence (GO:0090398)	46	0	0	-	< 0.01	1.00E+00	1.00E+00
**MUSCLE**							
negative regulation of cardiac muscle hypertrophy (GO:0010614)	26	1	0	+	> 100	1.31E-03	1.00E+00
negative regulation of muscle hypertrophy (GO:0014741)	28	1	0	+	> 100	1.41E-03	1.00E+00
regulation of cardiac muscle hypertrophy (GO:0010611)	54	1	0	+	> 100	2.67E-03	1.00E+00
regulation of muscle hypertrophy (GO:0014743)	57	1	0	+	> 100	2.82E-03	1.00E+00
regulation of muscle adaptation (GO:0043502)	82	1	0	+	> 100	4.03E-03	1.00E+00
regulation of muscle system process (GO:0090257)	231	1	0.01	+	89.16	1.13E-02	1.00E+00
positive regulation of cardiac muscle hypertrophy in response to stress (GO:1903244)	2	0	0	-	< 0.01	1.00E+00	1.00E+00
regulation of vascular associated smooth muscle contraction (GO:0003056)	9	0	0	-	< 0.01	1.00E+00	1.00E+00

© 2021. Cantu N et al. Published online at http://www.aginganddisease.org/EN/10.14336/AD.2021.0817

positive regulation of cardiac muscle contraction (GO:0060452)	8	0	0	-	< 0.01	1.00E+00	1.00E+00
muscle system process (GO:0003012)	288	0	0.01	-	< 0.01	1.00E+00	1.00E+00
involuntary skeletal muscle contraction (GO:0003011)	3	0	0	-	< 0.01	1.00E+00	1.00E+00
voluntary skeletal muscle contraction (GO:0003010)	3	0	0	-	< 0.01	1.00E+00	1.00E+00
skeletal muscle satellite cell migration (GO:1902766)	2	0	0	-	< 0.01	1.00E+00	1.00E+00
skeletal muscle contraction (GO:0003009)	30	0	0	-	< 0.01	1.00E+00	1.00E+00
muscle tissue morphogenesis (GO:0060415)	67	0	0	-	< 0.01	1.00E+00	1.00E+00
aorta smooth muscle tissue morphogenesis (GO:0060414)	3	0	0	-	< 0.01	1.00E+00	1.00E+00
positive regulation of growth factor dependent skeletal muscle satellite cell proliferation (GO:1902728)	2	0	0	-	< 0.01	1.00E+00	1.00E+00
positive regulation of skeletal muscle satellite cell proliferation (GO:1902724)	2	0	0	-	< 0.01	1.00E+00	1.00E+00
negative regulation of skeletal muscle satellite cell proliferation (GO:1902723)	4	0	0	-	< 0.01	1.00E+00	1.00E+00
regulation of striated muscle contraction (GO:0006942)	91	0	0	-	< 0.01	1.00E+00	1.00E+00
striated muscle contraction (GO:0006941)	107	0	0.01	-	< 0.01	1.00E+00	1.00E+00
regulation of smooth muscle contraction (GO:0006940)	60	0	0	-	< 0.01	1.00E+00	1.00E+00
smooth muscle contraction (GO:0006939)	49	0	0	-	< 0.01	1.00E+00	1.00E+00
regulation of muscle contraction (GO:0006937)	161	0	0.01	-	< 0.01	1.00E+00	1.00E+00
muscle contraction (GO:0006936)	241	0	0.01	-	< 0.01	1.00E+00	1.00E+00
cardiac muscle cell myoblast differentiation (GO:0060379)	10	0	0	-	< 0.01	1.00E+00	1.00E+00
regulation of ventricular cardiac muscle cell membrane depolarization (GO:0060373)	6	0	0	-	< 0.01	1.00E+00	1.00E+00
regulation of atrial cardiac muscle cell membrane repolarization (GO:0060372)	7	0	0	-	< 0.01	1.00E+00	1.00E+00
regulation of atrial cardiac muscle cell membrane depolarization (GO:0060371)	8	0	0	-	< 0.01	1.00E+00	1.00E+00
negative regulation of transcription from RNA polymerase II promoter involved in smooth muscle cell differentiation (GO:2000820)	3	0	0	-	< 0.01	1.00E+00	1.00E+00
regulation of ventricular cardiac muscle cell membrane repolarization (GO:0060307)	21	0	0	-	< 0.01	1.00E+00	1.00E+00
negative regulation of cardiac muscle cell differentiation (GO:2000726)	7	0	0	-	< 0.01	1.00E+00	1.00E+00
metanephric smooth muscle tissue development (GO:0072208)	2	0	0	-	< 0.01	1.00E+00	1.00E+00
regulation of cardiac muscle cell differentiation (GO:2000725)	16	0	0	-	< 0.01	1.00E+00	1.00E+00
positive regulation of cardiac muscle cell differentiation (GO:2000727)	9	0	0	-	< 0.01	1.00E+00	1.00E+00

© 2021. Cantu N et al. Published online at http://www.aginganddisease.org/EN/10.14336/AD.2021.0817

positive regulation of cardiac vascular smooth muscle cell differentiation (GO:2000724)	2	0	0	-	< 0.01	1.00E+00	1.00E+00
negative regulation of cardiac vascular smooth muscle cell differentiation (GO:2000723)	1	0	0	-	< 0.01	1.00E+00	1.00E+00
regulation of cardiac vascular smooth muscle cell differentiation (GO:2000722)	3	0	0	-	< 0.01	1.00E+00	1.00E+00
positive regulation of transcription from RNA polymerase II promoter involved in smooth muscle cell differentiation (GO:2000721)	2	0	0	-	< 0.01	1.00E+00	1.00E+00
positive regulation of cardiac muscle myoblast proliferation (GO:0110024)	3	0	0	-	< 0.01	1.00E+00	1.00E+00
regulation of cardiac muscle myoblast proliferation (GO:0110022)	3	0	0	-	< 0.01	1.00E+00	1.00E+00
kidney smooth muscle tissue development (GO:0072194)	2	0	0	-	< 0.01	1.00E+00	1.00E+00
ureter smooth muscle cell differentiation (GO:0072193)	3	0	0	-	< 0.01	1.00E+00	1.00E+00
ureter smooth muscle development (GO:0072191)	3	0	0	-	< 0.01	1.00E+00	1.00E+00
negative regulation of cardiac muscle cell myoblast differentiation (GO:2000691)	1	0	0	-	< 0.01	1.00E+00	1.00E+00
regulation of cardiac muscle cell myoblast differentiation (GO:2000690)	1	0	0	-	< 0.01	1.00E+00	1.00E+00
regulation of muscle filament sliding speed (GO:0032972)	2	0	0	-	< 0.01	1.00E+00	1.00E+00
regulation of muscle filament sliding (GO:0032971)	4	0	0	-	< 0.01	1.00E+00	1.00E+00
cardiac muscle thin filament assembly (GO:0071691)	3	0	0	-	< 0.01	1.00E+00	1.00E+00
striated muscle myosin thick filament assembly (GO:0071688)	3	0	0	-	< 0.01	1.00E+00	1.00E+00
positive regulation of smooth muscle cell chemotaxis (GO:0071673)	3	0	0	-	< 0.01	1.00E+00	1.00E+00
negative regulation of smooth muscle cell chemotaxis (GO:0071672)	4	0	0	-	< 0.01	1.00E+00	1.00E+00
regulation of smooth muscle cell chemotaxis (GO:0071671)	6	0	0	-	< 0.01	1.00E+00	1.00E+00
smooth muscle cell chemotaxis (GO:0071670)	2	0	0	-	< 0.01	1.00E+00	1.00E+00
positive regulation of tongue muscle cell differentiation (GO:2001037)	2	0	0	-	< 0.01	1.00E+00	1.00E+00
regulation of tongue muscle cell differentiation (GO:2001035)	2	0	0	-	< 0.01	1.00E+00	1.00E+00
positive regulation of skeletal muscle cell differentiation (GO:2001016)	6	0	0	-	< 0.01	1.00E+00	1.00E+00
negative regulation of skeletal muscle cell differentiation (GO:2001015)	6	0	0	-	< 0.01	1.00E+00	1.00E+00
regulation of skeletal muscle cell differentiation (GO:2001014)	20	0	0	-	< 0.01	1.00E+00	1.00E+00
relaxation of vascular associated smooth muscle (GO:0060087)	6	0	0	-	< 0.01	1.00E+00	1.00E+00
smooth muscle contraction involved in micturition (GO:0060083)	2	0	0	-	< 0.01	1.00E+00	1.00E+00
positive regulation of cardiac muscle cell proliferation (GO:0060045)	24	0	0	-	< 0.01	1.00E+00	1.00E+00
negative regulation of cardiac muscle cell proliferation (GO:0060044)	14	0	0	-	< 0.01	1.00E+00	1.00E+00

© 2021. Cantu N et al. Published online at http://www.aginganddisease.org/EN/10.14336/AD.2021.0817

cardiac muscle contraction (GO:0060048)	75	0	0	-	< 0.01	1.00E+00	1.00E+00
regulation of cardiac muscle cell proliferation (GO:0060043)	40	0	0	-	< 0.01	1.00E+00	1.00E+00
cardiac muscle cell proliferation (GO:0060038)	12	0	0	-	< 0.01	1.00E+00	1.00E+00
positive regulation of relaxation of cardiac muscle (GO:1901899)	2	0	0	-	< 0.01	1.00E+00	1.00E+00
negative regulation of relaxation of cardiac muscle (GO:1901898)	2	0	0	-	< 0.01	1.00E+00	1.00E+00
regulation of relaxation of cardiac muscle (GO:1901897)	6	0	0	-	< 0.01	1.00E+00	1.00E+00
positive regulation of muscle tissue development (GO:1901863)	23	0	0	-	< 0.01	1.00E+00	1.00E+00
negative regulation of muscle tissue development (GO:1901862)	11	0	0	-	< 0.01	1.00E+00	1.00E+00
regulation of muscle tissue development (GO:1901861)	41	0	0	-	< 0.01	1.00E+00	1.00E+00
extraocular skeletal muscle development (GO:0002074)	4	0	0	-	< 0.01	1.00E+00	1.00E+00
positive regulation of kidney smooth muscle cell differentiation (GO:2000358)	1	0	0	-	< 0.01	1.00E+00	1.00E+00
negative regulation of kidney smooth muscle cell differentiation (GO:2000357)	1	0	0	-	< 0.01	1.00E+00	1.00E+00
regulation of kidney smooth muscle cell differentiation (GO:2000356)	1	0	0	-	< 0.01	1.00E+00	1.00E+00
skeletal muscle acetylcholine-gated channel clustering (GO:0071340)	7	0	0	-	< 0.01	1.00E+00	1.00E+00
muscle cell proliferation (GO:0033002)	18	0	0	-	< 0.01	1.00E+00	1.00E+00
relaxation of smooth muscle (GO:0044557)	7	0	0	-	< 0.01	1.00E+00	1.00E+00
ventricular cardiac muscle cell membrane repolarization (GO:0099625)	12	0	0	-	< 0.01	1.00E+00	1.00E+00
atrial cardiac muscle cell membrane repolarization (GO:0099624)	5	0	0	-	< 0.01	1.00E+00	1.00E+00
positive regulation of vascular associated smooth muscle cell differentiation involved in phenotypic switching (GO:1905932)	1	0	0	-	< 0.01	1.00E+00	1.00E+00
regulation of cardiac muscle cell membrane repolarization (GO:0099623)	25	0	0	-	< 0.01	1.00E+00	1.00E+00
regulation of smooth muscle cell-matrix adhesion (GO:2000097)	4	0	0	-	< 0.01	1.00E+00	1.00E+00
negative regulation of cardiac muscle cell contraction (GO:0106135)	1	0	0	-	< 0.01	1.00E+00	1.00E+00
positive regulation of cardiac muscle cell contraction (GO:0106134)	1	0	0	-	< 0.01	1.00E+00	1.00E+00
negative regulation of skeletal muscle tissue regeneration (GO:0043417)	1	0	0	-	< 0.01	1.00E+00	1.00E+00
regulation of skeletal muscle tissue regeneration (GO:0043416)	8	0	0	-	< 0.01	1.00E+00	1.00E+00
positive regulation of skeletal muscle tissue regeneration (GO:0043415)	6	0	0	-	< 0.01	1.00E+00	1.00E+00
smooth muscle tissue development (GO:0048745)	19	0	0	-	< 0.01	1.00E+00	1.00E+00
negative regulation of muscle organ development (GO:0048635)	11	0	0	-	< 0.01	1.00E+00	1.00E+00
regulation of muscle organ development (GO:0048634)	43	0	0	-	< 0.01	1.00E+00	1.00E+00

© 2021. Cantu N et al. Published online at http://www.aginganddisease.org/EN/10.14336/AD.2021.0817

positive regulation of skeletal muscle tissue growth (GO:0048633)	4	0	0	-	< 0.01	1.00E+00	1.00E+00
negative regulation of skeletal muscle tissue growth (GO:0048632)	2	0	0	-	< 0.01	1.00E+00	1.00E+00
regulation of skeletal muscle tissue growth (GO:0048631)	6	0	0	-	< 0.01	1.00E+00	1.00E+00
skeletal muscle tissue growth (GO:0048630)	4	0	0	-	< 0.01	1.00E+00	1.00E+00
muscle organ morphogenesis (GO:0048644)	74	0	0	-	< 0.01	1.00E+00	1.00E+00
positive regulation of skeletal muscle tissue development (GO:0048643)	20	0	0	-	< 0.01	1.00E+00	1.00E+00
negative regulation of skeletal muscle tissue development (GO:0048642)	4	0	0	-	< 0.01	1.00E+00	1.00E+00
positive regulation of skeletal muscle fiber development (GO:0048743)	9	0	0	-	< 0.01	1.00E+00	1.00E+00
regulation of skeletal muscle fiber development (GO:0048742)	13	0	0	-	< 0.01	1.00E+00	1.00E+00
negative regulation of smooth muscle cell proliferation (GO:0048662)	51	0	0	-	< 0.01	1.00E+00	1.00E+00
positive regulation of smooth muscle cell proliferation (GO:0048661)	89	0	0	-	< 0.01	1.00E+00	1.00E+00
regulation of smooth muscle cell proliferation (GO:0048660)	142	0	0.01	-	< 0.01	1.00E+00	1.00E+00
negative regulation of uterine smooth muscle contraction (GO:0070473)	1	0	0	-	< 0.01	1.00E+00	1.00E+00
regulation of uterine smooth muscle contraction (GO:0070472)	10	0	0	-	< 0.01	1.00E+00	1.00E+00
uterine smooth muscle contraction (GO:0070471)	1	0	0	-	< 0.01	1.00E+00	1.00E+00
positive regulation of membrane depolarization during cardiac muscle cell action potential (GO:1900827)	1	0	0	-	< 0.01	1.00E+00	1.00E+00
negative regulation of membrane depolarization during cardiac muscle cell action potential (GO:1900826)	1	0	0	-	< 0.01	1.00E+00	1.00E+00
regulation of membrane depolarization during cardiac muscle cell action potential (GO:1900825)	4	0	0	-	< 0.01	1.00E+00	1.00E+00
skeletal muscle fiber development (GO:0048741)	24	0	0	-	< 0.01	1.00E+00	1.00E+00
cardiac muscle tissue development (GO:0048738)	168	0	0.01	-	< 0.01	1.00E+00	1.00E+00
positive regulation of ureter smooth muscle cell differentiation (GO:2000063)	1	0	0	-	< 0.01	1.00E+00	1.00E+00
negative regulation of ureter smooth muscle cell differentiation (GO:2000062)	1	0	0	-	< 0.01	1.00E+00	1.00E+00
regulation of ureter smooth muscle cell differentiation (GO:2000061)	1	0	0	-	< 0.01	1.00E+00	1.00E+00
regulation of ventricular cardiac muscle cell action potential (GO:0098911)	12	0	0	-	< 0.01	1.00E+00	1.00E+00
regulation of atrial cardiac muscle cell action potential (GO:0098910)	4	0	0	-	< 0.01	1.00E+00	1.00E+00
membrane repolarization during ventricular cardiac muscle cell action potential (GO:0098915)	12	0	0	-	< 0.01	1.00E+00	1.00E+00
membrane repolarization during atrial cardiac muscle cell action potential (GO:0098914)	5	0	0	-	< 0.01	1.00E+00	1.00E+00

© 2021. Cantu N et al. Published online at http://www.aginganddisease.org/EN/10.14336/AD.2021.0817

membrane depolarization during atrial cardiac muscle cell action potential (GO:0098912)	3	0	0	-	< 0.01	1.00E+00	1.00E+00
positive regulation of canonical Wnt signaling pathway involved in cardiac muscle cell fate commitment (GO:1901297)	1	0	0	-	< 0.01	1.00E+00	1.00E+00
regulation of cardiac muscle cell action potential involved in regulation of contraction (GO:0098909)	8	0	0	-	< 0.01	1.00E+00	1.00E+00
regulation of slow-twitch skeletal muscle fiber contraction (GO:0031449)	2	0	0	-	< 0.01	1.00E+00	1.00E+00
positive regulation of fast-twitch skeletal muscle fiber contraction (GO:0031448)	2	0	0	-	< 0.01	1.00E+00	1.00E+00
regulation of fast-twitch skeletal muscle fiber contraction (GO:0031446)	2	0	0	-	< 0.01	1.00E+00	1.00E+00
negative regulation of canonical Wnt signaling pathway involved in cardiac muscle cell fate commitment (GO:1901296)	1	0	0	-	< 0.01	1.00E+00	1.00E+00
regulation of cardiac muscle cell action potential (GO:0098901)	26	0	0	-	< 0.01	1.00E+00	1.00E+00
smooth muscle cell proliferation (GO:0048659)	4	0	0	-	< 0.01	1.00E+00	1.00E+00
regulation of skeletal muscle tissue development (GO:0048641)	26	0	0	-	< 0.01	1.00E+00	1.00E+00
positive regulation of muscle organ development (GO:0048636)	22	0	0	-	< 0.01	1.00E+00	1.00E+00
skeletal muscle adaptation (GO:0043501)	12	0	0	-	< 0.01	1.00E+00	1.00E+00
muscle adaptation (GO:0043500)	32	0	0	-	< 0.01	1.00E+00	1.00E+00
regulation of canonical Wnt signaling pathway involved in cardiac muscle cell fate commitment (GO:1901295)	2	0	0	-	< 0.01	1.00E+00	1.00E+00
positive regulation of smooth muscle cell-matrix adhesion (GO:1905609)	1	0	0	-	< 0.01	1.00E+00	1.00E+00
relaxation of skeletal muscle (GO:0090076)	3	0	0	-	< 0.01	1.00E+00	1.00E+00
relaxation of muscle (GO:0090075)	22	0	0	-	< 0.01	1.00E+00	1.00E+00
positive regulation of uterine smooth muscle contraction (GO:0070474)	7	0	0	-	< 0.01	1.00E+00	1.00E+00
positive regulation of uterine smooth muscle relaxation (GO:1900721)	1	0	0	-	< 0.01	1.00E+00	1.00E+00
regulation of uterine smooth muscle relaxation (GO:1900719)	1	0	0	-	< 0.01	1.00E+00	1.00E+00
detection of muscle stretch (GO:0035995)	7	0	0	-	< 0.01	1.00E+00	1.00E+00
response to muscle stretch (GO:0035994)	23	0	0	-	< 0.01	1.00E+00	1.00E+00
positive regulation of vascular associated smooth muscle cell apoptotic process (GO:1905461)	6	0	0	-	< 0.01	1.00E+00	1.00E+00
relaxation of cardiac muscle (GO:0055119)	14	0	0	-	< 0.01	1.00E+00	1.00E+00
negative regulation of cardiac muscle contraction (GO:0055118)	4	0	0	-	< 0.01	1.00E+00	1.00E+00
regulation of cardiac muscle contraction (GO:0055117)	74	0	0	-	< 0.01	1.00E+00	1.00E+00

© 2021. Cantu N et al. Published online at http://www.aginganddisease.org/EN/10.14336/AD.2021.0817

negative regulation of vascular associated smooth muscle cell apoptotic process (GO:1905460)	2	0	0	-	< 0.01	1.00E+00	1.00E+00
negative regulation of cardiac muscle tissue development (GO:0055026)	2	0	0	-	< 0.01	1.00E+00	1.00E+00
positive regulation of cardiac muscle tissue development (GO:0055025)	1	0	0	-	< 0.01	1.00E+00	1.00E+00
regulation of cardiac muscle tissue development (GO:0055024)	4	0	0	-	< 0.01	1.00E+00	1.00E+00
positive regulation of cardiac muscle tissue growth (GO:0055023)	32	0	0	-	< 0.01	1.00E+00	1.00E+00
negative regulation of cardiac muscle tissue growth (GO:0055022)	24	0	0	-	< 0.01	1.00E+00	1.00E+00
regulation of cardiac muscle tissue growth (GO:0055021)	58	0	0	-	< 0.01	1.00E+00	1.00E+00
regulation of vascular associated smooth muscle cell apoptotic process (GO:1905459)	1	0	0	-	< 0.01	1.00E+00	1.00E+00
positive regulation of striated muscle cell differentiation (GO:0051155)	47	0	0	-	< 0.01	1.00E+00	1.00E+00
positive regulation of muscle cell differentiation (GO:0051149)	76	0	0	-	< 0.01	1.00E+00	1.00E+00
negative regulation of muscle cell differentiation (GO:0051148)	58	0	0	-	< 0.01	1.00E+00	1.00E+00
negative regulation of striated muscle cell differentiation (GO:0051154)	34	0	0	-	< 0.01	1.00E+00	1.00E+00
regulation of striated muscle cell differentiation (GO:0051153)	86	0	0	-	< 0.01	1.00E+00	1.00E+00
positive regulation of smooth muscle cell differentiation (GO:0051152)	13	0	0	-	< 0.01	1.00E+00	1.00E+00
negative regulation of smooth muscle cell differentiation (GO:0051151)	16	0	0	-	< 0.01	1.00E+00	1.00E+00
regulation of cardiac muscle fiber development (GO:0055018)	1	0	0	-	< 0.01	1.00E+00	1.00E+00
cardiac muscle tissue growth (GO:0055017)	24	0	0	-	< 0.01	1.00E+00	1.00E+00
ventricular cardiac muscle cell development (GO:0055015)	11	0	0	-	< 0.01	1.00E+00	1.00E+00
atrial cardiac muscle cell development (GO:0055014)	3	0	0	-	< 0.01	1.00E+00	1.00E+00
cardiac muscle cell development (GO:0055013)	57	0	0	-	< 0.01	1.00E+00	1.00E+00
atrial cardiac muscle tissue morphogenesis (GO:0055009)	6	0	0	-	< 0.01	1.00E+00	1.00E+00
cardiac muscle tissue morphogenesis (GO:0055008)	59	0	0	-	< 0.01	1.00E+00	1.00E+00
cardiac muscle cell differentiation (GO:0055007)	81	0	0	-	< 0.01	1.00E+00	1.00E+00
regulation of skeletal muscle fiber differentiation (GO:1902809)	4	0	0	-	< 0.01	1.00E+00	1.00E+00
positive regulation of skeletal muscle fiber differentiation (GO:1902811)	3	0	0	-	< 0.01	1.00E+00	1.00E+00
skeletal muscle organ development (GO:0060538)	134	0	0.01	-	< 0.01	1.00E+00	1.00E+00
muscle tissue development (GO:0060537)	301	0	0.01	-	< 0.01	1.00E+00	1.00E+00
skeletal muscle tissue development (GO:0007519)	123	0	0.01	-	< 0.01	1.00E+00	1.00E+00
myoblast fate determination (GO:0007518)	2	0	0	-	< 0.01	1.00E+00	1.00E+00
muscle organ development (GO:0007517)	283	0	0.01	-	< 0.01	1.00E+00	1.00E+00
muscle cell fate determination (GO:0007521)	2	0	0	-	< 0.01	1.00E+00	1.00E+00

© 2021. Cantu N et al. Published online at http://www.aginganddisease.org/EN/10.14336/AD.2021.0817

visceral muscle development (GO:0007522)	1	0	0	-	< 0.01	1.00E+00	1.00E+00
somatic muscle development (GO:0007525)	3	0	0	-	< 0.01	1.00E+00	1.00E+00
cardiac muscle tissue regeneration (GO:0061026)	4	0	0	-	< 0.01	1.00E+00	1.00E+00
positive regulation of membrane repolarization during ventricular cardiac muscle cell action potential (GO:1905026)	2	0	0	-	< 0.01	1.00E+00	1.00E+00
negative regulation of membrane repolarization during ventricular cardiac muscle cell action potential (GO:1905025)	1	0	0	-	< 0.01	1.00E+00	1.00E+00
regulation of membrane repolarization during ventricular cardiac muscle cell action potential (GO:1905024)	3	0	0	-	< 0.01	1.00E+00	1.00E+00
response to electrical stimulus involved in regulation of muscle adaptation (GO:0014878)	3	0	0	-	< 0.01	1.00E+00	1.00E+00
response to muscle inactivity involved in regulation of muscle adaptation (GO:0014877)	10	0	0	-	< 0.01	1.00E+00	1.00E+00
response to injury involved in regulation of muscle adaptation (GO:0014876)	1	0	0	-	< 0.01	1.00E+00	1.00E+00
response to stimulus involved in regulation of muscle adaptation (GO:0014874)	16	0	0	-	< 0.01	1.00E+00	1.00E+00
cell growth involved in cardiac muscle cell development (GO:0061049)	9	0	0	-	< 0.01	1.00E+00	1.00E+00
positive regulation of cell growth involved in cardiac muscle cell development (GO:0061051)	8	0	0	-	< 0.01	1.00E+00	1.00E+00
regulation of cell growth involved in cardiac muscle cell development (GO:0061050)	18	0	0	-	< 0.01	1.00E+00	1.00E+00
negative regulation of cell growth involved in cardiac muscle cell development (GO:0061052)	10	0	0	-	< 0.01	1.00E+00	1.00E+00
muscle structure development (GO:0061061)	473	0	0.02	-	< 0.01	1.00E+00	1.00E+00
negative regulation of striated muscle tissue development (GO:0045843)	10	0	0	-	< 0.01	1.00E+00	1.00E+00
positive regulation of striated muscle tissue development (GO:0045844)	22	0	0	-	< 0.01	1.00E+00	1.00E+00
ureter smooth muscle contraction (GO:0014849)	2	0	0	-	< 0.01	1.00E+00	1.00E+00
urinary tract smooth muscle contraction (GO:0014848)	9	0	0	-	< 0.01	1.00E+00	1.00E+00
esophagus smooth muscle contraction (GO:0014846)	2	0	0	-	< 0.01	1.00E+00	1.00E+00
growth factor dependent regulation of skeletal muscle satellite cell proliferation (GO:0014843)	1	0	0	-	< 0.01	1.00E+00	1.00E+00
regulation of skeletal muscle satellite cell proliferation (GO:0014842)	13	0	0	-	< 0.01	1.00E+00	1.00E+00
skeletal muscle satellite cell proliferation (GO:0014841)	2	0	0	-	< 0.01	1.00E+00	1.00E+00
muscle hypertrophy (GO:0014896)	32	0	0	-	< 0.01	1.00E+00	1.00E+00
smooth muscle hypertrophy (GO:0014895)	1	0	0	-	< 0.01	1.00E+00	1.00E+00

© 2021. Cantu N et al. Published online at http://www.aginganddisease.org/EN/10.14336/AD.2021.0817

response to denervation involved in regulation of muscle adaptation (GO:0014894)	10	0	0	-	< 0.01	1.00E+00	1.00E+00
striated muscle atrophy (GO:0014891)	8	0	0	-	< 0.01	1.00E+00	1.00E+00
positive regulation of muscle hyperplasia (GO:0014739)	1	0	0	-	< 0.01	1.00E+00	1.00E+00
regulation of muscle hyperplasia (GO:0014738)	3	0	0	-	< 0.01	1.00E+00	1.00E+00
positive regulation of muscle atrophy (GO:0014737)	2	0	0	-	< 0.01	1.00E+00	1.00E+00
negative regulation of muscle atrophy (GO:0014736)	2	0	0	-	< 0.01	1.00E+00	1.00E+00
regulation of muscle atrophy (GO:0014735)	4	0	0	-	< 0.01	1.00E+00	1.00E+00
skeletal muscle hypertrophy (GO:0014734)	1	0	0	-	< 0.01	1.00E+00	1.00E+00
regulation of skeletal muscle adaptation (GO:0014733)	13	0	0	-	< 0.01	1.00E+00	1.00E+00
skeletal muscle atrophy (GO:0014732)	7	0	0	-	< 0.01	1.00E+00	1.00E+00
positive regulation of cardiac muscle cell apoptotic process (GO:0010666)	13	0	0	-	< 0.01	1.00E+00	1.00E+00
regulation of cardiac muscle cell apoptotic process (GO:0010665)	35	0	0	-	< 0.01	1.00E+00	1.00E+00
negative regulation of striated muscle cell apoptotic process (GO:0010664)	26	0	0	-	< 0.01	1.00E+00	1.00E+00
positive regulation of striated muscle cell apoptotic process (GO:0010663)	13	0	0	-	< 0.01	1.00E+00	1.00E+00
regulation of striated muscle cell apoptotic process (GO:0010662)	38	0	0	-	< 0.01	1.00E+00	1.00E+00
positive regulation of muscle cell apoptotic process (GO:0010661)	26	0	0	-	< 0.01	1.00E+00	1.00E+00
branchiomeric skeletal muscle development (GO:0014707)	3	0	0	-	< 0.01	1.00E+00	1.00E+00
striated muscle tissue development (GO:0014706)	286	0	0.01	-	< 0.01	1.00E+00	1.00E+00
skeletal muscle satellite cell activation (GO:0014719)	5	0	0	-	< 0.01	1.00E+00	1.00E+00
positive regulation of satellite cell activation involved in skeletal muscle regeneration (GO:0014718)	2	0	0	-	< 0.01	1.00E+00	1.00E+00
regulation of satellite cell activation involved in skeletal muscle regeneration (GO:0014717)	2	0	0	-	< 0.01	1.00E+00	1.00E+00
regulation of the force of skeletal muscle contraction (GO:0014728)	3	0	0	-	< 0.01	1.00E+00	1.00E+00
regulation of twitch skeletal muscle contraction (GO:0014724)	4	0	0	-	< 0.01	1.00E+00	1.00E+00
regulation of skeletal muscle contraction by calcium ion signaling (GO:0014722)	5	0	0	-	< 0.01	1.00E+00	1.00E+00
twitch skeletal muscle contraction (GO:0014721)	3	0	0	-	< 0.01	1.00E+00	1.00E+00
positive regulation of muscle adaptation (GO:0014744)	7	0	0	-	< 0.01	1.00E+00	1.00E+00
positive regulation of muscle hypertrophy (GO:0014742)	29	0	0	-	< 0.01	1.00E+00	1.00E+00
negative regulation of muscle hyperplasia (GO:0014740)	2	0	0	-	< 0.01	1.00E+00	1.00E+00
vascular associated smooth muscle cell development (GO:0097084)	10	0	0	-	< 0.01	1.00E+00	1.00E+00
myoblast migration involved in skeletal muscle regeneration (GO:0014839)	4	0	0	-	< 0.01	1.00E+00	1.00E+00

© 2021. Cantu N et al. Published online at http://www.aginganddisease.org/EN/10.14336/AD.2021.0817

myoblast differentiation involved in skeletal muscle regeneration (GO:0014835)	1	0	0	-	< 0.01	1.00E+00	1.00E+00
skeletal muscle satellite cell maintenance involved in skeletal muscle regeneration (GO:0014834)	5	0	0	-	< 0.01	1.00E+00	1.00E+00
positive regulation of smooth muscle cell apoptotic process (GO:0034393)	13	0	0	-	< 0.01	1.00E+00	1.00E+00
negative regulation of smooth muscle cell apoptotic process (GO:0034392)	7	0	0	-	< 0.01	1.00E+00	1.00E+00
regulation of smooth muscle cell apoptotic process (GO:0034391)	22	0	0	-	< 0.01	1.00E+00	1.00E+00
left ventricular cardiac muscle tissue morphogenesis (GO:0003220)	2	0	0	-	< 0.01	1.00E+00	1.00E+00
right ventricular cardiac muscle tissue morphogenesis (GO:0003221)	3	0	0	-	< 0.01	1.00E+00	1.00E+00
ventricular cardiac muscle tissue development (GO:0003229)	55	0	0	-	< 0.01	1.00E+00	1.00E+00
atrial cardiac muscle tissue development (GO:0003228)	8	0	0	-	< 0.01	1.00E+00	1.00E+00
post-embryonic cardiac muscle cell growth involved in heart morphogenesis (GO:0003247)	1	0	0	-	< 0.01	1.00E+00	1.00E+00
cardiac muscle tissue growth involved in heart morphogenesis (GO:0003245)	2	0	0	-	< 0.01	1.00E+00	1.00E+00
muscle hypertrophy in response to stress (GO:0003299)	14	0	0	-	< 0.01	1.00E+00	1.00E+00
physiological muscle hypertrophy (GO:0003298)	9	0	0	-	< 0.01	1.00E+00	1.00E+00
lung smooth muscle development (GO:0061145)	2	0	0	-	< 0.01	1.00E+00	1.00E+00
negative regulation of muscle contraction (GO:0045932)	22	0	0	-	< 0.01	1.00E+00	1.00E+00
positive regulation of muscle contraction (GO:0045933)	43	0	0	-	< 0.01	1.00E+00	1.00E+00
smooth muscle adaptation (GO:0014805)	4	0	0	-	< 0.01	1.00E+00	1.00E+00
smooth muscle hyperplasia (GO:0014806)	2	0	0	-	< 0.01	1.00E+00	1.00E+00
regulation of skeletal muscle contraction by regulation of release of sequestered calcium ion (GO:0014809)	4	0	0	-	< 0.01	1.00E+00	1.00E+00

© 2021. Cantu N et al. Published online at http://www.aginganddisease.org/EN/10.14336/AD.2021.0817
